# Cyclin A–CDK1 suppresses the expression of the CDK1 activator CDC25A to safeguard timely mitotic entry

**DOI:** 10.1016/j.jbc.2023.102957

**Published:** 2023-01-28

**Authors:** Lau Yan Ng, Hoi Tang Ma, Randy Y.C. Poon

**Affiliations:** 1 Division of Life Science, The Hong Kong University of Science and Technology, Hong Kong, China; 2 Department of Pathology, The University of Hong Kong, Hong Kong, China; 3 State Key Laboratory of Liver Research, The University of Hong Kong, Hong Kong, China; 4 State Key Laboratory of Molecular Neuroscience, The Hong Kong University of Science and Technology, Hong Kong, China

**Keywords:** cell cycle, cyclin, cyclin-dependent kinases, mitosis, phosphatase, AID, auxin-induced degron, CDK, cyclin-dependent kinases, CHX, cycloheximide, DI, Dox and IAA, Dox, doxycycline, IAA, indole-3-acetic acid, IR, ionizing radiation, NOC, nocodazole, tTA, tetracycline-controlled transcriptional activator

## Abstract

Cyclin A and CDC25A are both activators of cyclin-dependent kinases (CDKs): cyclin A acts as an activating subunit of CDKs and CDC25A a phosphatase of the inhibitory phosphorylation sites of the CDKs. In this study, we uncovered an inverse relationship between the two CDK activators. As cyclin A is an essential gene, we generated a conditional silencing cell line using a combination of CRISPR-Cas9 and degron-tagged cyclin A. Destruction of cyclin A promoted an acute accumulation of CDC25A. The increase of CDC25A after cyclin A depletion occurred throughout the cell cycle and was independent on cell cycle delay caused by cyclin A deficiency. Moreover, we determined that the inverse relationship with cyclin A was specific for CDC25A and not for other CDC25 family members or kinases that regulate the same sites in CDKs. Unexpectedly, the upregulation of CDC25A was mainly caused by an increase in transcriptional activity instead of a change in the stability of the protein. Reversing the accumulation of CDC25A severely delayed G_2_–M in cyclin A-depleted cells. Taken together, these data provide evidence of a compensatory mechanism involving CDC25A that ensures timely mitotic entry at different levels of cyclin A.

The cell cycle is choreographed by an evolutionarily conserved engine composed of a family of protein kinases called cyclin-dependent kinases (CDKs) (1). The current paradigm states that in human cells, CDK1 is activated by the mitotic cyclins (cyclin A and B) and drives G_2_ cells into mitosis (2). Another CDK family member, CDK2, associates mainly with cyclin E and cyclin A, and the complexes formed are critical for G_1_-S transition and in S phase, respectively (3). CDK4 and CDK6 are partners of cyclin D, functioning in G_1_–S transition before cyclin E–CDK2 (4).

The activities of CDKs are stringently regulated by protein–protein interactions and phosphorylation. For example, binding to a mitotic cyclin subunit is necessary for full activation of CDK1. On binding to cyclin B, the kinase activity of CDK1 is initially suppressed by inhibitory phosphorylation on CDK1^T14/^^Y^^15^ by MYT1 and WEE1 (5). At the end of G_2_, the stockpile of inactive cyclin B–CDK1 complexes is activated by members of the CDC25 dual-specificity phosphatase family (6). Active CDK1 then activates more CDC25 and inactivates WEE1 by directly phosphorylating these proteins. This autocatalytic loop enables rapid and complete activation of all the cyclin B–CDK1 complexes by an initially small amount of active CDK1.

The presence of three isoforms of CDC25 (A, B, and C) differing in cell cycle regulation, localization, and mood of regulation suggests that they may play nonoverlapping roles in the cell cycle. CDC25A appears to be particularly important, at least in mice, as knockout of Cdc25A results in early embryonic lethality (7). By contrast, mice lacking both Cdc25B and Cdc25C are generally normal (8). The prevailing view is that while CDC25B and CDC25C regulate mainly the G_2_–M cyclin–CDK complexes, CDC25A is involved in the control of both G_1_–S and G_2_–M cyclin–CDK pairs (9). Another unique feature of CDC25A distinguishing it from other isoforms is its rapid degradation in response to DNA damage or stalled replication forks. This mechanism is dependent on the ATM/ATR–CHK1/CHK2 pathway and is critical for the checkpoints that halt the cell cycle in response to genotoxic stresses (10).

Expression of CDC25A is periodically regulated during the cell cycle by both transcription and proteolysis. Transcription starting from late G_1_ is mediated by transcription factors including MYC and E2F (11, 12, 13). Unlike CDC25C or CDC25B, which is expressed throughout the cell cycle (14) or is targeted to proteasome-dependent degradation during mitosis (15), respectively, CDC25A accumulates during mitosis in a phosphorylated state (16). CDC25A is then targeted to ubiquitin-mediated degradation by APC/C^CDH1^ during mitotic exit and by SCF^βTrCP^ during interphase (17, 18, 19). DNA damage enhances the SCF^βTrCP^-mediated degradation of CDC25A through phosphorylation by CHK1/CHK2 (20, 21).

Similar to CDC25A, cyclin A also functions at multiple points in the cell cycle. During S phase, phosphorylation of various components of the prereplicative complexes by cyclin A–CDK2 complexes is involved in both the firing of DNA replication origins as well as preventing the re-firing of the same origins within the same cell cycle (22). Cyclin A also functions during G_2_–M, but its precise role is less well-defined. One hypothesis is that cyclin A itself is a component of M phase-promoting factor, the engine that drives cells into mitosis. An alternative hypothesis is that cyclin A is part of the network that triggers the activation of M phase-promoting factor (2, 6). For example, cyclin A–CDK has been implicated in turning on PLK1, which then activates CDC25C to allow cyclin B–CDK1 activation (23, 24).

In this study, we uncovered an additional relationship between cyclin A and CDC25A. Downregulation of cyclin A induced an accumulation of CDC25A through an increase of transcription. We provide evidence of a compensatory mechanism that ensure timely entry into mitosis at varying levels of cyclin A.

## Results

### Depletion of cyclin A triggers rapid accumulation of CDC25A

**Figure 1:**
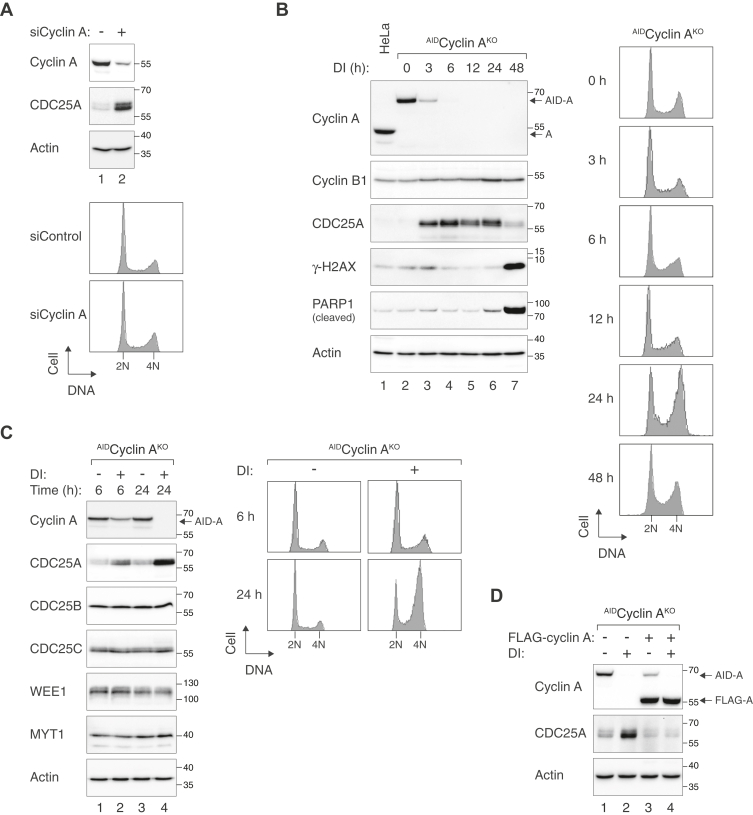
**Depletion of cyclin A triggers rapid accumulation of CDC25A.***A*, downregulation of cyclin A with siRNA induces accumulation of CDC25A. HeLa cells were transfected with either control or cyclin A siRNA. After 24 h, the cells were harvested and analyzed with immunoblotting (*upper panel*). Actin analysis was included to assess protein loading and transfer. The positions of molecular size standards (in kDa) are indicated on the *right*. The cells were also harvested for flow cytometry (*lower panel*). The positions of 2N and 4N DNA contents are indicated. *B*, conditional depletion of cyclin A stimulates rapid accumulation of CDC25A. HeLa cells lacking endogenous cyclin A and expressing AID-cyclin A (^AID^Cyclin A^KO^) were generated. The cells were cultured in the presence or absence of Dox and IAA (DI) to turn off AID-cyclin A before harvested at different time points for immunoblotting analysis (*left panel*) or flow cytometry analysis (*right panel*). Lysates of control HeLa cells were included to indicate the position and abundance of endogenous cyclin A. Cleaved PARP1 and γ-H2AX are markers of apoptosis and DNA damage, respectively. *C*, depletion of cyclin A specifically induces CDC25A but not the other CDK^T14/Y15^ regulators. ^AID^Cyclin A^KO^ cells were either untreated or treated with DI and harvested at the indicated time points. The cells were analyzed with immunoblotting (*left panel*) and flow cytometry (*right panel*). *D*, ectopic expression of cyclin A suppresses cyclin A^KO^-induced CDC25A accumulation. ^AID^Cyclin A^KO^ cells were transiently transfected with either control or FLAG-cyclin A expression plasmid. A blasticidin resistance–expressing plasmid was cotransfected for transient selection for 36 h to enrich transfected cells. At 24 h after removing the blasticidin-containing medium, the cells were treated with or without DI for 6 h before harvested for immunoblotting analysis. AID, auxin-induced degron; Dox, doxycycline; IAA, indole-3-acetic acid.

We initially found that downregulation of cyclin A in HeLa cells with siRNA promoted an accumulation of CDC25A (Fig. 1*A*). It was noteworthy that siRNA-mediated depletion of cyclin A was not highly effective, and cell cycle distribution was not significantly altered after transfection (Fig. 1*A*, lower panel). This suggested that the striking increase of CDC25A did not require complete depletion of cyclin A or changes in cell cycle distribution.

Given the limitations associated with siRNAs, including their slow kinetics, incomplete knockdown, and nonspecificity, we next generated a conditional cell line for acute and tight silencing of cyclin A based on a recently developed tetracycline-controlled transcriptional activator (tTA)–auxin-induced degron (AID) dual transcription–degron system (25, 26). Concurrent with the disruption of cyclin A with CRISPR-Cas9, an AID-tagged cDNA of cyclin A (which was resistant to the CRISPR-Cas9 due to the introduction of silence mutations at the CRISPR-Cas9-targeting site) under the control of a Tet-Off promoter was integrated into the genome. Transcription of the cDNA by tTA could be turned off using doxycycline (Dox). Moreover, AID-tagged cyclin A could be targeted to rapid proteolysis when indole-3-acetic acid (IAA) was added.

Figure 1*B* shows that cells lacking endogenous cyclin A and expressing AID-cyclin A (designated as ^AID^Cyclin A^KO^ herein) were able to degrade AID-cyclin A effectively in response to Dox and IAA (DI) treatment, in effect producing a cyclin A-deficient environment. The AID-cyclin A was expressed to a similar level as the endogenous cyclin A (before CRISPR-Cas9-mediated disruption) and was destroyed rapidly after DI addition. Quantifying the band intensity (using a serially diluted standard curve) revealed that ∼5% of AID-cyclin A remained at 6 h (and ∼1% at 9 h) after DI treatment (Fig. S1). The destruction of AID-cyclin A was accompanied with a rapid and sustained accumulation of CDC25A (Fig. 1*B*). In some experiments, the expression of CDC25A decreased at later time points (48 h), probably due to the increase in DNA damage and apoptosis after prolonged cyclin A depletion (indicated by the accumulation of γ-H2AX and cleaved PARP1, respectively).

Turning off cyclin A resulted in delays in both S and G_2_/M (Figs. 1*B* and S2*A*). As CDC25A accumulated before substantial alteration of cell cycle distribution occurred, it is unlikely that the increase of CDC25A was an indirect outcome of cell cycle redistribution. This was validated later using synchronized cells (see below). Furthermore, the increase of CDC25A after cyclin A destruction was confirmed using other independently isolated clones of ^AID^Cyclin A^KO^, indicating that it was not a consequence of clonal effects (Fig. S2*B*). Unlike CDC25A, other CDK1^T14/Y15^ kinases and phosphatases including CDC25B, CDC25C, WEE1, and MYT1 were unaffected by cyclin A depletion (Fig. 1*C*).

Although the ^AID^Cyclin A^KO^ cell line was a rescue system by design, we further transfected a FLAG-tagged cyclin A into the cells before degrading AID-cyclin A. Figure 1*D* shows that ectopically expressed FLAG-cyclin A could reverse the accumulation of CDC25A. Although the expression of CDC25A was generally low in control cells, overexpression of cyclin A was able to further reduce CDC25A expression. Furthermore, we also generated ^AID^Cyclin A^KO^ in H1299 cells and found that CDC25A was enriched in the absence of cyclin A, indicating that the phenomenon was not limited to HeLa cells (Fig. S2*C*).

**Figure 2:**
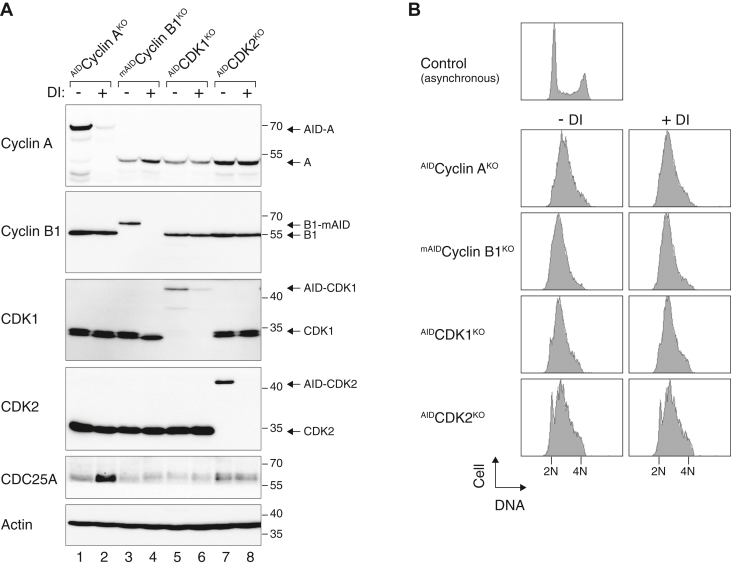
**Depletion of cyclin A but not cyclin B1, CDK1, or CDK2 results in CDC25A accumulation.** Specificity of cyclin A depletion–mediated CDC25A accumulation. Cells from ^AID^Cyclin A^KO^, ^mAID^Cyclin B1^KO^, ^AID^CDK1^KO^, and ^AID^CDK2^KO^ were synchronized with a double thymidine block procedure. The cells were then cultured in fresh or DI-containing medium. *A* and *B*, after 6 h, the cells were harvested and analyzed with (*A*) immunoblotting and (*B*) flow cytometry. AID, auxin-induced degron; DI, Dox and IAA.

Finally, using a similar approach as ^AID^Cyclin A^KO^, we generated inducible depletion of cell lines expressing AID (or mini-AID)-tagged cyclin B1, CDK1, and CDK2 in backgrounds lacking the respective endogenous genes. To avoid cell cycle–related effects on CDC25A, the cells were first synchronized in S phase with a double thymidine block before incubated with DI for 6 h to turn off the AID/mAID proteins. By contrast to cyclin A, depletion of cyclin B1, CDK1, or CDK2 during S phase did not result in CDC25A accumulation (Fig. 2), indicating a specific role of cyclin A in this process.

Collectively, these results revealed that depletion of cyclin A promotes the rapid and specific accumulation of CDC25A.

### Cyclin A regulates CDC25A throughout the cell cycle

One of the difficulties in studying cyclin A is owing to the multifaceted functions of cyclin A during the cell cycle (functioning at both S phase and mitosis and binding to both CDK1 and CDK2). Moreover, CDC25A expression is also highly regulated during the cell cycle (10). To exclude the possibility that the accumulation of CDC25A triggered by cyclin A depletion was caused by a disruption of the cell cycle, we next examined the effect of cyclin A destruction at different periods of the cell cycle using synchronized samples.

**Figure 3:**
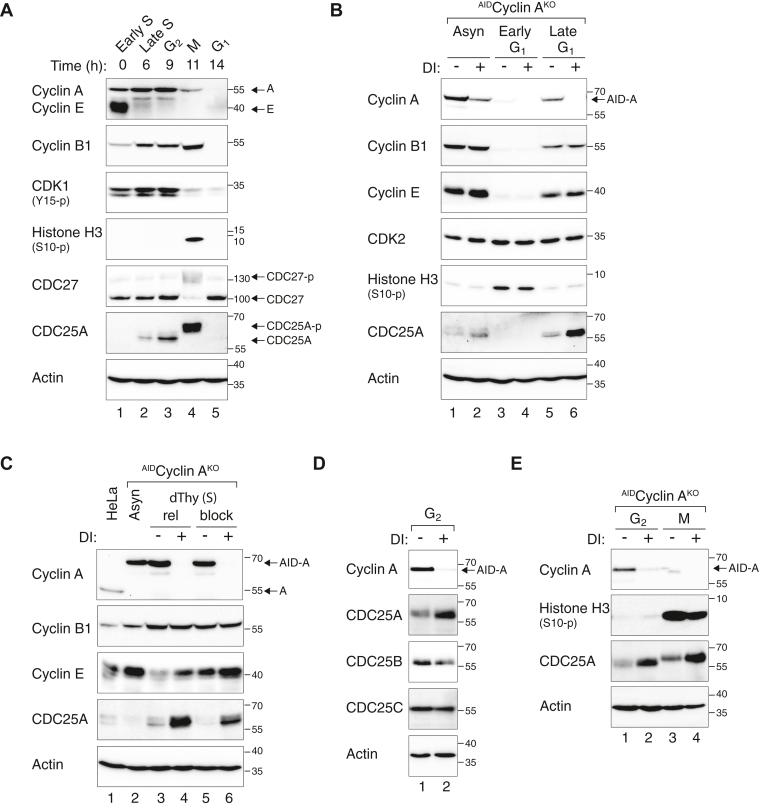
**Cyclin A regulates CDC25A throughout the cell cycle.***A*, CDC25A expression starts at late S, accumulates in G_2_, and degrades at the end of mitosis. HeLa cells were synchronized using a double thymidine block procedure. Cells at different cell phases of the cell cycle were harvested at the indicated time points after release from the block. NOC was added to the mitotic sample (M) for 6 h before cells were isolated with mechanical shake-off. A portion of the mitotic cells were released from the block by washing and re-plating in NOC-free medium for 3 h before harvested as G_1_ cells. Lysates were prepared and analyzed with immunoblotting. Histone H3^S10^ phosphorylation and CDC27 mobility shifts are mitotic markers. Dephosphorylation of CDK1^Y15^ occurred as cells entered mitosis. Note that CDC25A was phosphorylated and displayed a gel mobility shift during mitosis. The DNA contents of the cells were also examined with flow cytometry to validate the synchronization (Fig. S3*A*). *B*, preventing cyclin A accumulation during late G_1_ promotes CDC25A accumulation. ^AID^Cyclin A^KO^ cells were synchronized in mitosis using a NOC block procedure as described in Experimental procedures. The cells were then released into NOC-free medium for 3 h and 6 h to obtain cells in early and late G_1_, respectively. The cells were treated with or without DI for 3 h before harvested. Asynchronized cells were included for comparison. Cell-free extracts were prepared and analyzed with immunoblotting. The synchronization was also assessed with flow cytometry (Fig. S3*B*). *C*, destruction of cyclin A promotes CDC25A accumulation during S phase. ^AID^Cyclin A^KO^ cells were synchronized at early S phase with a double thymidine block procedure. The cells were then mock- or DI-treated, either after released from the block or continually incubated with thymidine. After 6 h, the cells were harvested and analyzed with immunoblotting. Flow cytometry analysis of the samples are shown in Fig. S3*C*. *D*, loss of cyclin A specifically increases CDC25A but not CDC25B or CDC25C in G_2_ cells. After synchronization with a double thymidine block, ^AID^Cyclin A^KO^ cells were released into drug-free or DI-containing medium. After 9 h, G_2_ cells were obtained for immunoblotting analysis. Flow cytometry analysis of the samples are shown in Fig. S3*D*. *E*, destruction of cyclin A promotes CDC25A accumulation during both G_2_ and mitosis. ^AID^Cyclin A^KO^ cells were synchronized with a double thymidine block and released into drug-free or DI-containing medium. NOC was added after 4 h. G_2_ and mitotic cells were isolated as described in panel *A*. Flow cytometry analysis of the samples are shown in Fig. S3*E*. AID, auxin-induced degron; DI, Dox and IAA; NOC, nocodazole.

We first confirmed the robust cell cycle variation of CDC25A in synchronized HeLa cells (Fig. 3*A*). As expected, CDC25A expression was relatively low in early S phase and started to accumulate in S and G_2_. It was highly phosphorylated during mitosis and became undetectable in G_1_, consistent with its APC/C^CDH1^-dependent proteolysis (17).

As both cyclin A and CDC25A were undetectable in early G_1_, it is not surprising that turning off cyclin A during that time did not affect CDC25A expression (Fig. 3*B*). However, destruction of cyclin A in late G_1_ was already able to promote an accumulation of CDC25A (Fig. 3*B*). Likewise, cells synchronously released into S phase using a double thymidine block procedure also contained increased amount of CDC25A after cyclin A was turned off (Fig. 3*C*). It is noteworthy that CDC25A was also increased when cyclin A was destroyed in cells continuously blocked with thymidine, indicating that cell cycle progression was not required for cyclin A-mediated CDC25A accumulation. This suggested that any cell cycle delay caused by cyclin A depletion was not required for CDC25A accumulation. In fact, as cyclin A depletion delayed S and G_2_/M (Fig. S2*A*), it is expected that CDC25A level should become lower (instead of higher as observed) if cell cycle effects caused by cyclin A depletion plays a major role in CDC25A expression. Finally, turning off cyclin A in synchronized G_2_ (Fig. 3*D*) or mitotic (Fig. 3*E*) cells also promoted CDC25A accumulation.

Taken together, these data indicate that the increase of CDC25A after cyclin A depletion occurs throughout the cell cycle and is independent on cell cycle delays caused by cyclin A deficiency.

### Cyclin A regulates CDC25A independently to DNA damage-mediated degradation

**Figure 4:**
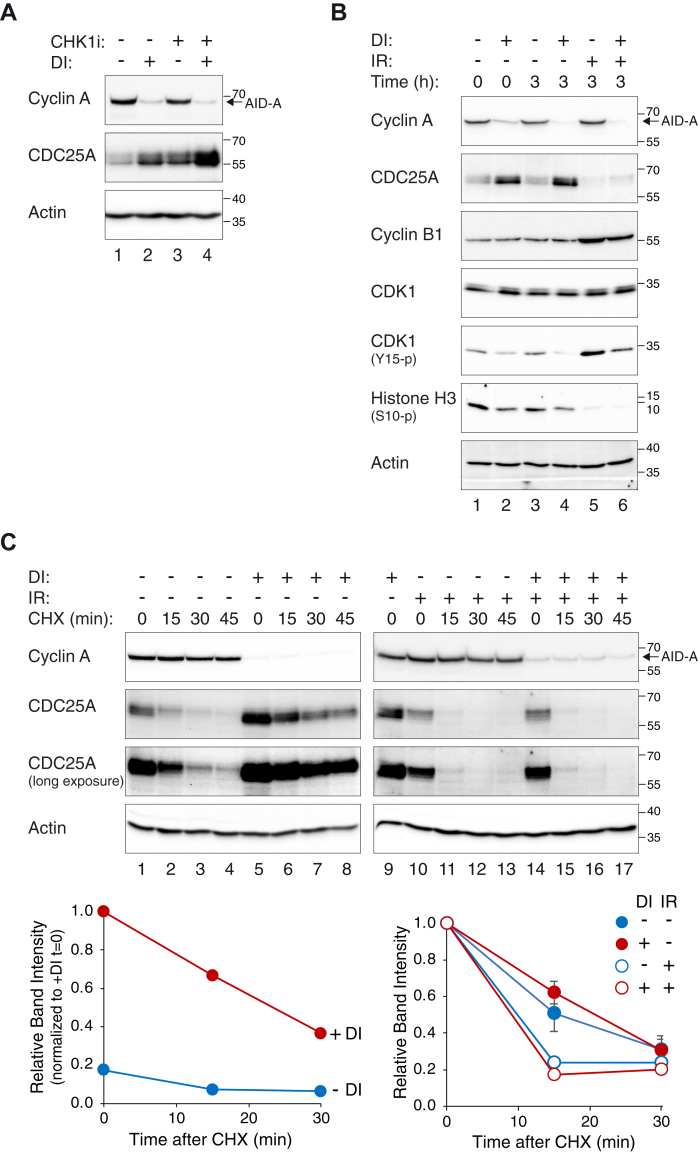
**Cyclin A regulates CDC25A independently to DNA damage–mediated degradation.***A*, depletion of cyclin A triggers CDC25A accumulation independently of CHK1 pathway. ^AID^Cyclin A^KO^ cells were incubated in the presence or absence of DI and/or AZD7762 (CHK1i) for 6 h. Lysates were prepared and analyzed with immunoblotting. *B*, cyclin A-deficient cells remain susceptible to DNA damage–mediated CDC25A degradation. ^AID^Cyclin A^KO^ cells were incubated with DI for 6 h to turn off AID-cyclin A before irradiated with 15 Gy of IR. The cells were harvested either immediately or after 3 h. Cell-free extracts were prepared and analyzed with immunoblotting. *C*, cyclin A depletion does not increase CDC25A protein stability. ^AID^Cyclin A^KO^ cells were pretreated with DI for 6 h to turn off AID-cyclin A. The cells were then either mock-treated or irradiated with 15 Gy of IR. After 3 h, cycloheximide (CHX) was added to abolish *de novo* protein synthesis. At the indicated time points after CHX addition, the cells were harvested and the abundance of CDC25A was determined by immunoblotting. The same DI-treated sample (lanes 5 and 9) was included in different gels for normalization. The relative CDC25A band intensity was quantified using densitometry and serially diluted standard curves (not shown) and normalized either to +DI (t = 0) (*lower left-hand panel*) or to t = 0 of individual samples (*lower right-hand panel*; mean ± SEM of two independent experiments). AID, auxin-induced degron; DI, Dox and IAA.

A major mechanism of CDC25A regulation during the cell cycle is through SCF^βTrCP^-mediated degradation, which can be accelerated by CHK1 (20, 27). As shown previously (28), inhibition of CHK1 using a small chemical inhibitor AZD7762 (CHK1i) stabilized CDC25A (Fig. 4*A*). Nevertheless, CDC25A was further induced after cyclin A was turned off, suggesting that the increase of CDC25A upon cyclin A depletion was independent on the SCF^βTrCP^ pathway. Furthermore, the CDC25A in cyclin A-depleted cells could be targeted to degradation after ionizing radiation (IR), suggesting the DNA damage-mediated CDC25A degradation mechanism remained intact in the absence of cyclin A (Fig. 4*B*). CDC25A was rapidly degraded to a background level after irradiation irrespective of the presence or absence of cyclin A (Fig. S4*A*). Finally, we also examined other substrates of SCF^βTrCP^ and APC/C^CDH1^ after cyclin A degradation (29). Unlike CDC25A, the expression of several substrates of SCF^βTrCP^ (including WEE1 (Fig. 1*C*) and EMI1 (Fig. S4*B*)) or APC/C^CDH1^ (including cyclin B1 (Fig. 1*B*) and PLK1 (Fig. S4*B*)) was unaffected by cyclin A depletion. Collectively, these data indicate that the increase of CDC25A after cyclin A destruction is not caused by inhibiting SCF^βTrCP^- or APC/C^CDH1^-dependent turnover.

### Depletion of cyclin A increases transcription but not protein stability of CDC25A

**Figure 5:**
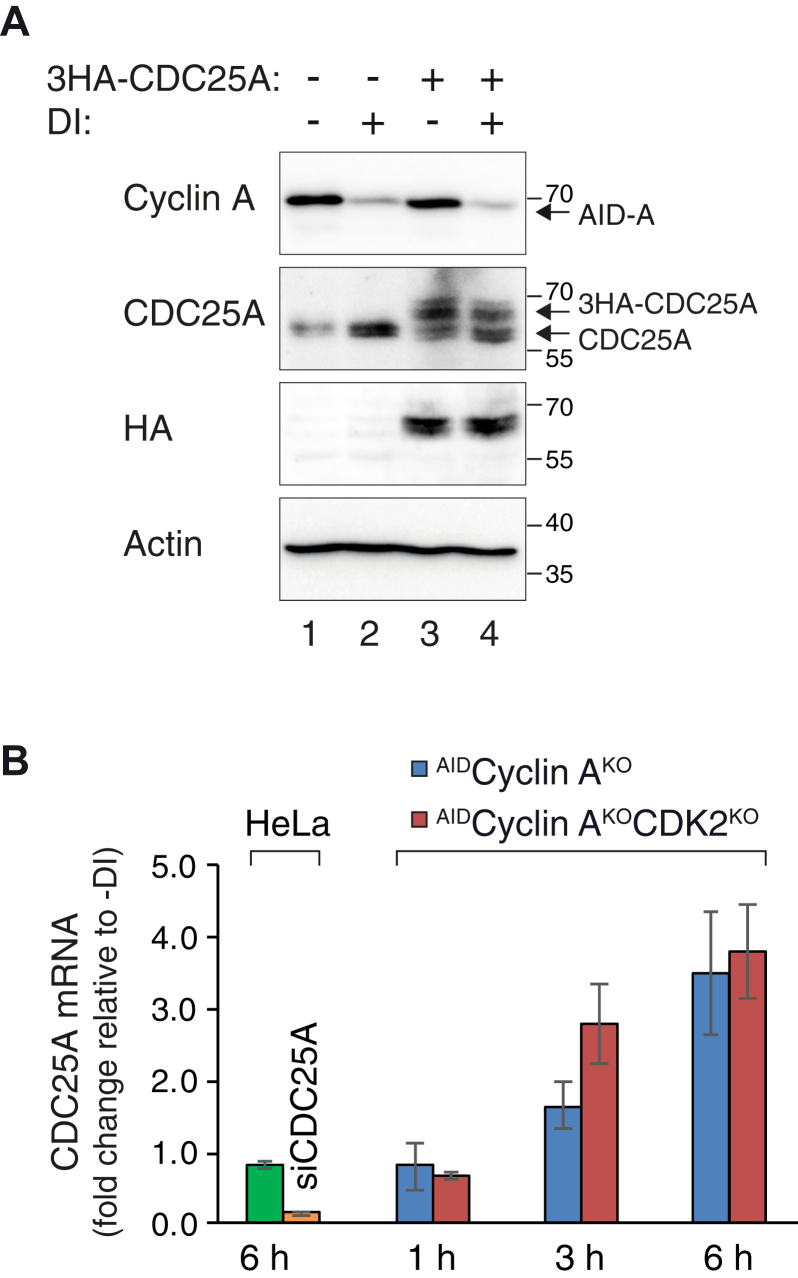
**Cyclin A transcriptionally downregulates CDC25A.***A*, expression of exogenous CDC25A is independent of cyclin A. ^AID^Cyclin A^KO^ cells were transiently transfected with control or a plasmid expressing 3HA-CDC25A under a constitutive promoter. A plasmid expressing a blasticidin-resistant gene was cotransfected. At 24 h after transfection, transfected cells were enriched by selection with blasticidin for 36 h. After recovery in normal medium for 24 h, the cells were incubated with DI for 6 h to turn off AID-cyclin A. Lysates were prepared and analyzed with immunoblotting. *B*, depletion of cyclin A increases CDC25A mRNA level. HeLa, ^AID^Cyclin A^KO^, and ^AID^Cyclin A^KO^CDK2^KO^ cells were cultured in the presence or absence of DI for the indicated time before harvested for RNA extraction. As a control, HeLa cells were transfected with CDC25A siRNA (siCDC25A) for 30 h before harvested. Reverse transcription and quantitative real-time PCR were performed using primers against CDC25A. Primers to actin were used as a normalization control (mean ± SEM of three independent experiments). The data were normalized to -DI for the individual time point. AID, auxin-induced degron; DI, Dox and IAA.

We next examined the protein stability of CDC25A by using cycloheximide (CHX) to abolish *de novo* protein synthesis. Although CDC25A was expressed at a higher level before the addition of CHX in cyclin A-depleted cells than in control cells, its protein stability was not significantly increased after cyclin A was depleted (Fig. 4*C*). The half-lives of CDC25A in both cyclin A-containing and -deficient cells were ∼17.6 min after the addition of CHX. In agreement with the above results, IR-induced DNA damage further reduced the stability of CDC25A in both cyclin A-containing and -deficient environments. Unlike that of the endogenous CDC25A, the expression of exogenous CDC25A driven by a constitutive promoter was unaltered after the destruction of cyclin A (Fig. 5*A*). As a control, both endogenous and exogenous CDC25A could be stabilized with a CHK1 inhibitor (Figs. 4*A* and S4*C*). This provided further evidence that the regulation of CDC25A by cyclin A was not caused by a change in protein stability.

Given that the protein stability of CDC25A was not affected by cyclin A, we next investigated if the expression of CDC25A mRNA was affected by cyclin A. Figure 5*B* shows that CDC25A mRNA accumulated after the destruction of cyclin A in a time-dependent manner. Similar results were obtained using an ^AID^Cyclin A^KO^ cell line that also lacked CDK2, which displayed a stronger G_2_–M delay than cells lacking cyclin A alone (manuscript in preparation). Transcriptome analysis using RNA sequencing confirmed that CDC25A transcript was upregulated after cyclin A was destroyed in G_2_-synchronized cells (Fig. S5). By contrast, CDC25B and CDC25C transcripts were downregulated in the absence of cyclin A.

Taken together, these results indicate that the increase in CDC25A after downregulation of cyclin A was mainly caused by an increase in transcriptional activity instead of a change in protein stability.

### Regulation of CDC25A by cyclin A ensures timely mitotic entry

**Figure 6:**
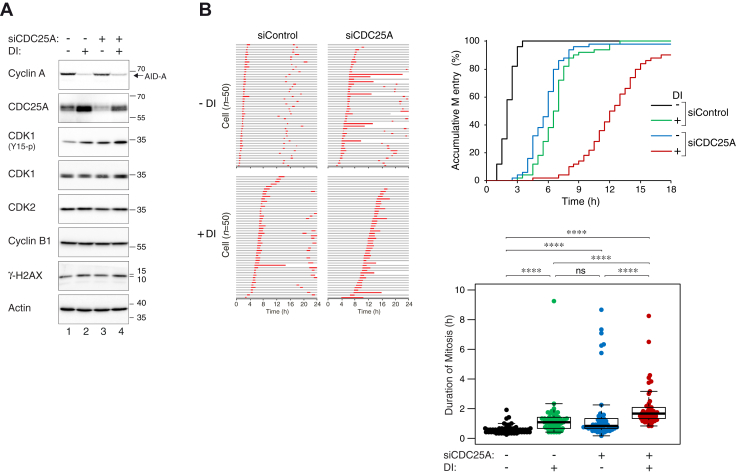
**Cyclin A and CDC25A synergistically drive cells into mitosis.***A*, partial knockdown of CDC25A in ^AID^Cyclin A^KO^ cells. ^AID^Cyclin A^KO^ cells were synchronized by double thymidine block and transfected with either control or CDC25A siRNA (siCDC25A) at the time of the first thymidine release. At the second thymidine release, the cells were treated with DI for 7 h before subjected to live-cell imaging analysis. Cells collected at 3 h after the start of live-cell imaging were analyzed with immunoblotting. A concentration of CDC25A siRNA (250 pM) was used that was able to reduce CDC25A to a level similar to that before cyclin A was depleted. *B*, CDC25A is involved in ensuring timely mitotic entry in cyclin A-depleted cells. Cells were transfected and synchronized as described in panel *A*. Individual cells were then tracked with time-lapse microscopy for 24 h. Each *horizontal bar* represents one cell (n = 50). *Gray*: interphase; *red*: mitosis (from cell rounding to anaphase B or telophase); *truncated bars*: cell death. The accumulative percentage of cells entering mitosis is plotted against time. Box-and-whisker plots show the duration of mitosis measured as time elapsed from onset of cell rounding to anaphase B (n = 50) (∗∗∗∗*p* < 0.0001; ns *p* > 0.05). AID, auxin-induced degron.

To evaluate the biological consequences of CDC25A accumulation in response to the downregulation of cyclin A, CDC25A was downregulated with siRNA, and mitotic entry was analyzed at single-cell level using live-cell imaging. We titrated the concentration of the siRNA so that CDC25A was reduced to a level similar to that before cyclin A was depleted (Fig. 6*A*). The cells were synchronously released into the cell cycle from a double thymidine block before turning off the cyclin A (Fig. 6*B*). As expected, mitotic entry was deferred after the destruction of cyclin A. Reducing CDC25A likewise delayed mitotic entry. Mitotic entry was further delayed when CDC25A was downregulated in cyclin A-depleted cells, suggesting that the normal accumulation of CDC25A in these cells was responsible for partially compensating for the loss of cyclin A. A similar delay in mitotic entry was observed after cyclin A and CDC25A were downregulated in cells synchronized in late G_2_ using the CDK1 inhibitor RO3306 (Fig. S6). In contrast to CDC25A, depletion of CDC25C did not delay G_2_–M in the presence or absence of cyclin A (Fig. S7).

In addition to the delay in G_2_–M, the duration of mitosis was also increased after downregulation of cyclin A and CDC25A (Fig. 6*B*). The delay in G_2_–M in cyclin A- and CDC25A-depleted cells was consistent with an elevated CDK1^Y15^ phosphorylation (Fig. 6*A*). As γ-H2AX was not stimulated significantly, the G_2_–M delay was unlikely to be caused by DNA damage associated with the experimental procedure.

Taken together, these data suggest a mechanism in which CDC25A is involved in compensating the alteration of cyclin A expression for controlling timely entry into mitosis.

## Discussion

Both cyclin A and CDC25A have been reasoned to play critical functions in controlling the cell cycle, albeit definitive evidence pinpointing their precise roles is generally lacking. For cyclin A, this is in part due to the presence of other cyclin–CDK pairs functioning at similar parts of the cell cycle (cyclin B–CDK and cyclin E–CDK for G_2_–M and G_1_–S, respectively) as well as the multiple functions of cyclin A. Likewise, the presence of CDC25B and CDC25C complicates the interpretations of experiments on CDC25A, which also functions in both G_2_–M and G_1_–S. This is compounded by the confusion of whether CDKs aside from CDK1 are actually regulated by inhibitory phosphorylation in different cell lines. For example, inhibitory phosphorylation plays a major role in the regulation of CDK1 but only a minor role for CDK2 during the unperturbed cell cycle of HeLa cells (30). However, replacing CDK2 with a nonphosphorylatable mutant of CDK2 in HCT116 accelerates S phase entry by several hours (31).

In this study, we found that downregulation of cyclin A with either siRNA (Fig. 1*A*) or CRISPR-Cas9 (in combination with a degron-mediated conditional depletion system, Figs. 1*B* and S2) promoted the accumulation of CDC25A. Conversely, overexpression of cyclin A reduced CDC25A (Fig. 1*D*). Interestingly, the increase of CDC25A was evident even when cyclin A was only partially depleted or before completely destroyed (see for example Fig. 1, *A* and *B*).

Although CDC25A expression is cell cycle regulated (Fig. 3*A*), several lines of evidence suggest that the cyclin A-mediated regulation of CDC25A was not solely due to cell cycle disruption. First, the kinetics of CDC25A accumulation was rapid, occurring before changes of cell cycle distribution was observed with flow cytometry (Fig. 1*B*). Second, CDC25A accumulated in cyclin A-deficient cells synchronized in late G_1_, S, and G_2_ (Fig. 3, *B*–*D*). Moreover, CDC25A was increased after cyclin A destruction even during a thymidine-induced S phase block (Fig. 3*C*). Finally, as depletion of cyclin A delayed interphase progression, as indicated by flow cytometry (Fig. 1*B*), BrdU incorporation (Fig. S2*A*), and live-cell imaging (Fig. 6*B*), it is expected that cyclin A depletion reduced instead of increased CDC25A if a cell cycle effect was involved.

Established mechanisms of CDC25A regulation by cyclin–CDK complexes center around phosphatase activity and protein turnover (10). Phosphorylation of Ser18 and Ser116 of CDC25A by cyclin B–CDK1 during mitosis stabilizes CDC25A (16). Phosphorylation of CDC25A’s Ser283 by cyclin–CDK at late S/G_2_ increases its G_2_–M-promoting activity without affecting its stability (32). On the other hand, cyclin D–CDK4/CDK6 complexes are implicated in destabilizing CDC25A by phosphorylating Ser40, which primes the phosphorylation of Ser88 for SCF^βTrCP^-dependent degradation (33). Inhibition of cyclin A–CDK2 was also found to increase CDC25A, albeit no direct evidence of a decrease of protein turnover was obtained (34). However, we found that the accumulation of CDC25A after cyclin A destruction was not associated with an increase in protein stability (Fig. 4*C*). This was further supported by the lack of stabilization of exogenously expressed CDC25A (Fig. 5*A*). Instead, the increase of CDC25A appears to be caused by an increase of CDC25A mRNA (Fig. 5*B*). This is consistent with results from whole transcriptome analysis (Fig. S5). It is noteworthy that the transcriptome analysis revealed that in addition to CDC25A mRNA, many transcripts were upregulated in the absence of cyclin A. Further investigation will be needed to establish whether the accumulation of transcripts of other genes also results in an increase at the protein level.

We do not yet understand the mechanisms of how cyclin A affects the transcription of CDC25A. The mechanism could be multifaceted because similar to many cell cycle regulators, CDC25A is controlled by a complex network at the transcriptional level. Well-established transcription factors for CDC25A include MYC (11) and E2F (12, 13). The CDC25A promoter possesses binding elements for SP1 and NF-Y (35, 36). Other transcription factors implicated in the control of CDC25A includes β-catenin (37), FOXM1 (38), NANOG (39), NPAS2 (40), and STAT3 (41). Several of these transcription factors have been shown to be directly regulated by cyclin A–CDK activities, including E2F (42, 43), SP1 (44, 45, 46), NY1 (47), FOXM1 (48), NANOG (49), and the APC–Axin–β-catenin pathway (50, 51, 52). In addition, it is possible that the large number of miRNAs reported in the literature that can regulate CDC25A may also be regulated by cyclin A.

Cyclin A affected the expression of specifically the CDC25A isoform. Several lines of evidence suggest that CDC25A is the most important isoform of the CDC25 family. There is evidence suggesting that centrosomally located CDC25B is an initiator of G_2_–M through its activation of the centrosomal subpopulation of cyclin B–CDK1, which is then able to initiate the autocatalytic loop to activate all the cyclin B–CDK1 (53). However, Cdc25B and Cdc25C are not required for mouse development (8). Furthermore, a case with homozygous deletion mutation of CDC25B in human also does not prevent live birth (with development of clinical defects including cataracts, dilated cardiomyopathy, and multiple endocrinopathies) (54). On the other hand, Cdc25A is essential for mouse development (7). Interestingly, similar to cyclin A, CDC25A has been implicated to play roles in both the G_1_–S and G_2_–M (9). Our data suggest that CDC25A is in a position to compensate for the variation of cyclin A during the cell cycle.

Both cyclin A and CDC25A were normally at their lowest levels in the cell cycle during G_1_. Hence it is not surprising that depletion of cyclin A (and the resulting increase in CDC25A) during G_1_ did not significantly affect the timing of S phase entry (our unpublished data). However, given that cyclin A is an integral component of the S phase-promoting engine, its downregulation is expected to promote replicative stress. In agreement with this hypothesis, double-strand breaks are generated after the loss of cyclin A (55). Likewise, there is evidence that overexpression of CDC25A can promote replicative stress by slowing down replication forks and inducing fork reversal (56). The resulting replication-derived DNA lesions are then carried into mitosis, causing chromosome segregation defects (57). Overexpression of CDC25A has been reported in various human cancer tissues (58). Overexpressed CDC25A can cooperate with oncogenes or loss of tumor suppressor genes in oncogenic transformation (59, 60). Hence downregulation of cyclin A and the consequent CDC25A accumulation are expected to act synergistically in promoting genome instability.

**Figure 7:**
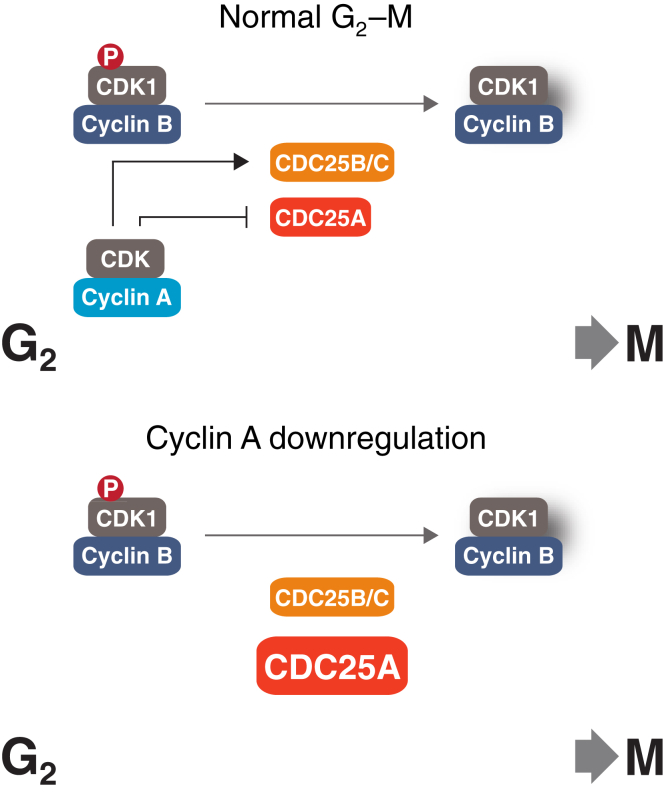
**A model of the role of cyclin A–CDK in modulating CDC25A level during G**_**2**_**-M.** During the G_2_-M transition, members of the CDC25 family antagonize WEE1 (and MYT1) by removing the inhibitory phosphorylation on cyclin B–CDK1 complexes. Cyclin A–CDK complexes are involved in the activation of CDC25B and CDC25C through the PLK1 pathway. We showed that cyclin A–CDK complexes contribute to transcriptional repression of CDC25A during interphase, preventing premature activation of cyclin B–CDK1. Downregulation of cyclin A removes this repression and promotes CDC25A accumulation. We postulate that the excess CDC25A acts as a compensatory mechanism for cyclin A-depleted cells to overcome the G_2_-M barrier by promoting cyclin B–CDK1 activation.

We hypothesize that when cyclin A is downregulated, an increase of CDC25A could counterbalance the lowering CDK activities by activating cyclin B–CDK complexes. Conversely, overexpressed cyclin A could be compensated by a downregulation of CDC25A to delay mitotic entry. This relatively simple mechanism may ensure that mitotic entry could be fine-tuned to occur at an optimal time in spite of different expression of cyclin A (see Fig. 7 for a model). In support of this hypothesis, live-cell imaging analysis revealed that both cyclin A and CDC25A were rate limiting for G_2_–M in synchronized cells (Fig. 6*B*). Moreover, mitotic entry was further delayed in cyclin A-deficient cells by reducing CDC25A to a level similar to before cyclin A was destroyed (Fig. 6*B*).

As CDC25A is implicated to play a critical role in the DNA damage checkpoints (10), another possible route that cyclin A deficiency may lead to genome instability is through the impairment of this checkpoint. However, as CDC25A could still be rapidly degraded after irradiation in cyclin A-deficient cells (Fig. S4*A*), it is unlikely that genome instability associated with cyclin A downregulation was contributed by the effects of CDC25A on the DNA damage checkpoint.

Collectively, our results indicate that transcription regulation of CDC25A by cyclin A may set a threshold of CDC25 for mitotic entry. This is coupled with other CDK-dependent activation of CDC25A including direct activation of phosphatase activity, indirect activation through upstream regulators such as PLK1, as well as stabilization of the protein.

## Experimental procedures

### Plasmids

CRISPR-Cas9 constructs targeting cyclin A (CAGTATGAGAGCTATCCTCG) or CDC25A (AAGAGCAGGCGGCGGCGGTG) were prepared by ligating the annealing products of 5′-CACCGCAGTATGAGAGCTATCCTCG-3′ and 5′-AAACCGAGGATAGCTCTCATACTGC-3′ or 5′-CACCGAAGAGCAGGCGGCGGCGGTG-3′ and 5′-AAACCACCGCCGCCGCCTGCTCTTC-3′, respectively, to BbsI-cut pX330 (a gift from Feng Zhang; obtained from Addgene; Addgene#42230). CDK2 CRISPR-Cas9 in pX330 was generated as previously described (25).

FLAG-3C-cyclin A in pCDNA3.1(−) was generated as previously described (61). CRISPR-resistant silent mutations were introduced into cyclin A with a double PCR procedure. In the first PCR, FLAG-cyclin A in pUHD-P3 (62) was used as a template and amplified with 5′-AGCTCGTTTAGTGAACCGTCAGATCG-3′ and 5′-GCCATATTGGTAGACTGGTTAGTTG-3′ and 5′-GTCTACCAATATGGCTCTCATACTG-3′ and 5′-TATCTTATCATGTCTGGATCC-3′. These PCR products were then amplified in the second PCR using the franking primers (first and last primers above). AID-cyclin A constructs were generated by inserting NcoI–BamHI-cut fragment of the double PCR product into NcoI–BamHI-cut pRevTRE-AID/Hyg (25) or pUHD-SB-AID/Hyg (26).

### siRNA

Stealth siRNA targeting cyclin A (GCUAUGCUGUUAGCCUCAAAGUUUG) and control siRNA were manufactured by Life Technologies. CDC25A siRNA (CCAACCUGACCGUCACUAUTT) and CDC25C siRNA (GCGGCUACAGAGACUUCUUTT) were manufactured by Shanghai Genepharma.

### Cell culture

Cells were propagated in Dulbecco’s modified Eagle’s medium supplemented with 10% (v/v) calf serum (for HeLa) or fetal bovine serum (for H1299) and 50 U/ml of penicillin streptomycin (Thermo Fisher Scientific).

Unless stated otherwise, cells were treated with the following reagents at the indicated final concentration: blasticidin (Thermo Fisher Scientific; 3.75 μg/ml for transient selection; 2.5 μg/ml for stable selection), CHK1i (AZD7762) (Selleck Chemicals; 20 nM), CHX (Sigma-Aldrich; 10 μg/ml), Dox (Sigma-Aldrich; 2 μg/ml), hygromycin B (Thermo Fisher Scientific; 0.25 mg/ml), IAA (Sigma-Aldrich; 50 μg/ml), nocodazole (NOC) (Sigma-Aldrich; 100 ng/ml), puromycin (Sigma-Aldrich; 0.75 μg/ml for transient selection; 0.3 μg/ml for stable selection), RO3306 (Santa Cruz Biotechnology; 10 μM), and thymidine (Santa Cruz Biotechnology; 2 mM). Cells were transfected with plasmids using a calcium phosphate precipitation method (63). Transfection of siRNA (10 nM) was carried out using Lipofectamine RNAiMAX (Thermo Fisher Scientific) according to manufacturer instructions. Unless stated otherwise, transfected cells were cultured for 24 h before harvested for further analysis.

### Cell lines

HeLa (cervical carcinoma) used in this study was a clone expressing the tTA tetracycline transactivator (64). H1299 cells were obtained from American Type Culture Collection. Cell lines ^AID^CDK1^KO^ (26) and ^AID^CDK2^KO^ (25) were generated as previously described. ^mAID^Cyclin B1^KO^ was a HeLa cell line expressing cyclin B1-mAID without endogenous cyclin B1 (Adrijana Crncec and RYCP, manuscript in preparation).

^AID^Cyclin A^KO^ cells from HeLa were generated by retroviral infection (25) using the construct AID-cyclin A in pRevTRE-AID/Hyg, followed by transfection of cyclin A CRISPR-Cas9 in pX330. ^AID^Cyclin A^KO^ cells from H1299 were generated by transfecting H1299 cells with AID-cyclin A in pUHD-SB-AID/Hyg, pSBbi-TIR1-tTA/Pur (26), cyclin A CRISPR-Cas9 in pX330, and Sleeping Beauty transposase (pCMV(CAT)T7-SB100; a gift from Zsuzsanna Izsvak; Addgene, #34879) before selecting with hygromycin and puromycin for 2 weeks. ^AID^Cyclin A^KO^ cells lacking CDK2 were generated by cotransfecting CDK2 CRISPR-Cas9 in pX330 and a plasmid expressing blasticidin-resistant gene (a gift from Tim Hunt, Cancer Research UK) into ^AID^Cyclin A^KO^ HeLa cells. After enriching the transfected cells with blasticidin selection for 36 h, the cells were recovered in blasticidin-free medium for 48 h. In all the above cell lines, single cell-derived colonies were obtained by limiting dilution in 96-well plates.

### Synchronization

Synchronization with double thymidine and NOC shake-off was performed as previously described (65). Briefly, cells were grown in medium containing 2 mM of thymidine for 14 h. The cells were then washed twice with PBS and cultured in fresh medium. After 9 h, the cells were incubated with a second round of 2 mM of thymidine for 14 h to obtain early S cells. Late S and G_2_ cells were obtained at 6 h and 9 h after release from the double thymidine block, respectively.

For synchronization using RO3306 blockade, cells were first synchronized using 2 mM of thymidine for 14 h. The cells were then washed twice with PBS and cultured in fresh medium for 6 h before incubation with RO3306 for another 6 h. After washed twice with PBS, the attached cells were harvested as late G_2_ cells.

For NOC shake-off synchronization, double thymidine-synchronized cells were released for 6 h before incubation with NOC for 6 h. Mitotic cells were collected by mechanical shake-off followed by centrifugation. G_1_ cells were obtained by washing the mitotic cells with PBS twice and released into drug-free medium. After 3 h, attached cells were harvested as G_1_ cells.

Double thymidine synchronization coupling with siRNA transfection was performed as described (66). In brief, cells were transfected with siRNA after the release from the first thymidine block. Fresh medium was then replenished before applying the second thymidine block. Cell-free extracts were prepared as described previously (67).

### Ionizing radiation

IR was delivered with a caesium-137 source from a Gammacell 1000 Elite Irradiator (Nordion).

### Live-cell imaging

Cells were seeded onto 24-well cell culture plates and placed into an automated microscopy system with temperature, humidity, and CO_2_ control chamber (Zeiss Celldiscoverer 7). Images were captured every 5 min for up to 24 h. Data acquisition was carried out with Zeiss ZEN 2.3 (blue edition), and analysis was performed using ImageJ (National Institutes of Health). After mitosis, one of the daughter cells was randomly selected and continued to be tracked.

### Flow cytometry

Flow cytometry analysis after propidium iodide staining was performed as previously described (68). Briefly, cells were trypsinized and washed with PBS. The cells were then fixed with ice-cold 70% ethanol and stained with a solution containing 40 μg/ml propidium iodide and 40 μg/ml RNase A at 37 °C for 30 min. DNA contents of 10,000 cells were analyzed with FACSAria III (BD Biosciences).

For BrdU incorporation analysis, cells were pulsed with 10 μM of BrdU for 30 min before harvesting. The cells were then fixed with ice-cold 80% ethanol. After centrifugation at 2000 rpm for 5 min, the pellet was washed twice with PBS before incubated with freshly made 2 M HCl at 25 °C for 20 min with gentle mixing. To neutralize the HCl, the cells were incubated with 0.1 M sodium borate buffer (pH 8.5) at 25 °C for 5 min. The cell pellet obtained after centrifugation were washed twice with PBS and once with PBST (PBS with 0.5% Tween 20 and 0.05% w/v BSA). The cell pellet was resuspended in residue buffer and incubated with 2 μl of anti-BrdU antibody (DAKO) at 25 °C for 1.5 h. The cells were then washed twice with PBST before incubating with 2 μl of Alexa Fluor-488 goat anti-mouse IgG antibody (Thermo Fisher Scientific) at 25 °C for 1 h in the dark. After washing twice with PBST, the cells were subjected to propidium iodide staining and flow cytometry analysis.

### Quantitative real-time PCR

Total RNA extraction, reverse transcription PCR, and real-time PCR were performed as previously described (25). Primers against CDC25A were: 5′-CCTCCGAGTCAACAGATTCA-3′ and 5′-GGGTCGATGAGCTGAAAGAT-3′. The expression of CDC25A mRNA was normalized to that of actin. Fold-change of sample normalized to control was calculated by 2^−ΔΔCt^ method.

### Transcriptome analysis

Biological replicates of G_2_ cells were harvested at 9 h after release from double thymidine synchronization. Immediately after harvesting, total RNA was extracted using NucleoSpin RNA kit (Macherey-Nagel). The samples were then air-dried in RNA stabilization tubes according to the manufacturer’s instructions before dispatched for library preparation and RNA-sequencing (Genewiz).

For data analysis, fastq files were aligned using STAR algorithm (version 2.5.2a) using *Homo sapiens* GRCh38.83 as the reference genome. Reads were then counted using HTSeq-Counts (69) prior to downstream analysis. Statistical analyses on read counts were performed using the DESeq2 package (70) to identify differentially expressed (DE) genes between two experimental groups (±DI). Genes exhibited a fold change >1 or adjusted *p* value <0.05 were considered DE. Volcano plots were generated using DEBrowser (version 1.10.9; (71)) using R (version 3.6.3; www.R-project.org) to visualize DE genes upon cyclin A depletion.

### Antibodies and immunological methods

The following antibodies were obtained from the indicated sources: monoclonal antibodies against beta-actin (Sigma-Aldrich); cyclin A2 (AT10, a gift from Tim Hunt, Cancer Research UK); cyclin B1 (sc-245, Santa Cruz Biotechnology), cyclin E1 (sc-247, Santa Cruz Biotechnology), CDK1 (sc-54, Santa Cruz Biotechnology), CDK2 (sc-6248, Santa Cruz Biotechnology), CDK1(Y15p) (612307, BD Biosciences), cleaved PARP1 (552597, BD Biosciences), CDC25A (sc-7389, Santa Cruz Biotechnology), CDC25B (ab124819, Abcam), CDC25C (sc-13138, Santa Cruz Biotechnology), WEE1 (sc-5285, Santa Cruz Biotechnology), CDC27 (610455, BD Biosciences), EMI1 (37-6600, Zymed Laboratories), PLK1 (sc-17783, Santa Cruz Biotechnology), polyclonal antibodies against phosphorylated histone H3^S10^ (sc-8656R, Santa Cruz Biotechnology), MYT1 (sc-13081, Santa Cruz Biotechnology), γ-H2AX (A300-081A, Bethyl Laboratories), and HA (A190-208A, Bethyl Laboratories). Immunoblotting was performed as previously described (25). The positions of molecular size standards (in kDa) are indicated in the Figures. Quantification of signals on immunoblotting was conducted with Image Lab software (version 5.2.1 build 11, Bio-Rad Laboratories).

### Statistical analysis

Box-and-whisker plots (center lines show the medians; box limits indicate interquartile range; whiskers extend to the most extreme data points that were no more than 1.5 times the interquartile range from the 25th and 75th percentiles) were generated using RStudio (version 1.3.1093). Mann–Whitney–Wilcoxon test was used to calculate statistical significance.

## Data availability

All primary data are available upon request.

## Supporting information

This article contains supporting information.

## Conflict of interest

The authors declare no conflict of interest with the contents of this article.

## Supporting information

Figure S1 **The kinetics of AID-cyclin A degradation.**^AID^Cyclin A^KO^ cells were treated with DI to turn off AID-cyclin A and harvested at the indicated time points. Lysates were prepared and analyzed with immunoblotting. Lysates from the parental HeLa cells were included to indicate the expression level of endogenous cyclin A. The signals of the AID-cyclin A were quantified using a standard curve from serial dilution of ^AID^Cyclin A^KO^ cell lysates (lanes 1–5) and plotted (*lower panel*). AID, auxin-induced degron; DI, Dox and IAA. **Figure S2. Depletion of cyclin A triggers rapid accumulation of CDC25A.***A*, cyclin A depletion delays the cell cycle in both S phase and G_2_/M progression. ^AID^Cyclin A^KO^ cells were cultured in the presence or absence of DI for 48 h before pulsed with BrdU for 30 min. The cells were fixed, stained with anti-BrdU antibody and propidium iodide, and analyzed with flow cytometry. The percentage of cells in different cell cycle phases was quantified (mean ± SEM of three independent experiments). *B*, destruction of cyclin A leads to CDC25A accumulation. Two independent clones of ^AID^Cyclin A^KO^ cells were cultured in the presence of DI and harvested at the indicated time points. The cells were then analyzed with immunoblotting (*upper panel*) and flow cytometry (*lower panel*). *C*, destruction of cyclin A promotes CDC25A accumulation in H1299 cells. ^AID^Cyclin A^KO^ cells were generated in H1299 cells. After 6 h of DI treatment, the cells were harvested for immunoblotting of cyclin A and CDC25A. The parental H1299 and ^AID^Cyclin A^KO^ in HeLa were included for comparison. AID, auxin-induced degron; DI, Dox and IAA. **Figure S3. Cyclin A regulates CDC25A throughout the cell cycle.***A*, cell cycle profiles of samples from the experiment described in Figure 3*A*. Cells were synchronized at different phases of the cell cycle after release from double thymidine block. The samples were fixed and analyzed with flow cytometry. *B*, cell cycle profiles of samples from the experiment described in Figure 3*B*. Cells were released from mitotic block in the presence or absence of DI. At early and late G_1_, the samples were fixed and analyzed with flow cytometry. Asynchronous cells were used as controls. *C*, cell cycle profiles of samples from the experiment described in Figure 3*C*. Cells were synchronized with a double thymidine block and either released into S phase or continually blocked with thymidine. After mock-treated or treated with DI, the samples were fixed and analyzed with flow cytometry. *D*, cell cycle profiles of samples from the experiment described in Figure 3*D*. Cells were synchronized with a double thymidine block and released in the presence or absence of DI. At G_2_, the samples were fixed and analyzed with flow cytometry. *E*, cell cycle profiles of samples from the experiment described in Figure 3*E*. Cells were synchronized with a double thymidine block and released in the presence or absence of DI. After incubation with NOC at different time points, G_2_ and mitotic (M) samples were obtained and analyzed with flow cytometry. DI, Dox and IAA; NOC, nocodazole. **Figure S4. Proteolysis of CDC25A is unaffected by the absence of cyclin A.***A*, DNA damage-mediated rapid CDC25A degradation is unaffected by the absence of cyclin A. ^AID^Cyclin A^KO^ cells were either mock-treated or incubated with DI for 6 h before irradiated with 15 Gy of IR. The expression of CDC25A at the different time points after IR was analyzed with immunoblotting; *B*, destruction of cyclin A increases CDC25A but not other targets of the βTrCP pathway. ^AID^Cyclin A^KO^ cells were grown in the presence or absence of DI before harvested at the indicated time points for immunoblotting analysis. *C*, ectopically expressed CDC25A is stabilized in the presence of AZD7762. HeLa cells were transiently transfected with control or a plasmid expressing 3HA-CDC25A under a constitutive promoter. At 48 h after transfection, the cells were incubated in the presence or absence of AZD7762 (CHK1i) for 5.5 h. Lysates were prepared and analyzed with immunoblotting. AID, auxin-induced degron; DI, Dox and IAA. **Figure S5. Transcriptomic analysis of cyclin AKO cells identified CDC25A as a differentially upregulated gene.** G_2_-enriched AIDCyclin A^KO^CDK2^KO^ cells were prepared and treated with DI as described in Methods. Total RNA extraction and RNA-sequencing were performed to obtain transcriptomic profiles comparing cyclin A-containing and deficient samples from two independent experiments. Differential gene expression is represented in a volcano plot. Genes identified as upregulated and downregulated are depicted in red and blue, respectively. *Gray* represents genes that are not significantly altered upon cyclin A disruption in G_2_ cells. The *inset* is the zoom-in region highlighting the transcriptomic alterations of CDC25A (upregulated), CDC25B and CDC25C (downregulated) in cyclin A-depleted cells. AID, auxin-induced degron; DI, Dox and IAA. **Figure S6. Cyclin A and CDC25A synergistically drive cells into mitosis.**^AID^Cyclin A^KO^ cells were transfected with either control or CDC25A siRNA (siCDC25A) at 9 h before thymidine block (for 14 h). After release from the thymidine block, the cells were treated with RO3306 for 6 h before treated with buffer or DI. After 1.5 h, the cells were released from the RO3306 block (DI was replenished) and subjected to live-cell imaging analysis. Each *horizontal bar* represents one cell (n = 50). *Gray*: interphase; *red*: mitosis (from cell rounding to anaphase B or telophase); truncated bars: cell death (*upper panel*). The accumulative percentage of cells entering mitosis is plotted against time (*lower panel*). AID, auxin-induced degron; DI, Dox and IAA. **Figure S7. CDC25A but not CDC25C is involved in ensuring timely mitotic entry in cyclin A-depleted cells.***A*, knockdown of CDC25C in ^AID^Cyclin A^KO^ cells. ^AID^Cyclin A^KO^ cells were synchronized by double thymidine block and transfected with either control or CDC25C siRNAs (10 nM) at the time of the first thymidine release. At the second thymidine release, the cells were treated with DI for 7 h before subjected to live-cell imaging. Cells collected at 3 h after the start of live-cell imaging were analyzed with immunoblotting. *B*, CDC25A but not CDC25C is involved in ensuring timely mitotic entry in cyclin A-depleted cells. Cells were transfected and synchronized as described in panel A. CDC25A siRNA was also transfected as a control. Individual cells were then tracked with time-lapse microscopy for 24 h. Each horizontal bar represents one cell (n = 50). *Gray*: interphase; *red*: mitosis (from cell rounding to anaphase B or telophase); *truncated bars*: cell death (*upper panel*). The accumulative percentage of cells entering mitosis is plotted against time (*lower panel*). AID, auxin-induced degron; DI, Dox and IAA.
