# Supplementary material for: Cyclin A–CDK1 suppresses the expression of the CDK1 activator CDC25A to safeguard timely mitotic entry
Source: J Biol Chem. 2023 Jan 28;299(3):102957. doi: 10.1016/j.jbc.2023.102957 (PMC9986519; doi:10.1016/j.jbc.2023.102957)
Supplement: Figure S1 — The kinetics of AID-cyclin A degradation.AIDCyclin AKO cells were treated with DI to turn off AID-cyclin A and harvested at the indicated time points. Lysates were prepared and analyzed with immunoblotting. Lysates from the parental HeLa cells were included to indicate the expression level of endogenous cyclin A. The signals of the AID-cyclin A were quantified using a standard curve from serial dilution of AIDCyclin AKO cell lysates (lanes 1–5) and plotted (lower panel). AID, auxin-induced degron; DI, Dox and IAA. Figure S2. Depletion of cyclin A triggers rapid accumulation of CDC25A.A, cyclin A depletion delays the cell cycle in both S phase and G2/M progression. AIDCyclin AKO cells were cultured in the presence or absence of DI for 48 h before pulsed with BrdU for 30 min. The cells were fixed, stained with anti-BrdU antibody and propidium iodide, and analyzed with flow cytometry. The percentage of cells in different cell cycle phases was quantified (mean ± SEM of three independent experiments). B, destruction of cyclin A leads to CDC25A accumulation. Two independent clones of AIDCyclin AKO cells were cultured in the presence of DI and harvested at the indicated time points. The cells were then analyzed with immunoblotting (upper panel) and flow cytometry (lower panel). C, destruction of cyclin A promotes CDC25A accumulation in H1299 cells. AIDCyclin AKO cells were generated in H1299 cells. After 6 h of DI treatment, the cells were harvested for immunoblotting of cyclin A and CDC25A. The parental H1299 and AIDCyclin AKO in HeLa were included for comparison. AID, auxin-induced degron; DI, Dox and IAA. Figure S3. Cyclin A regulates CDC25A throughout the cell cycle.A, cell cycle profiles of samples from the experiment described in Figure 3A. Cells were synchronized at different phases of the cell cycle after release from double thymidine block. The samples were fixed and analyzed with flow cytometry. B, cell cycle profiles of samples from the experiment described in F [file mmc1.pdf]

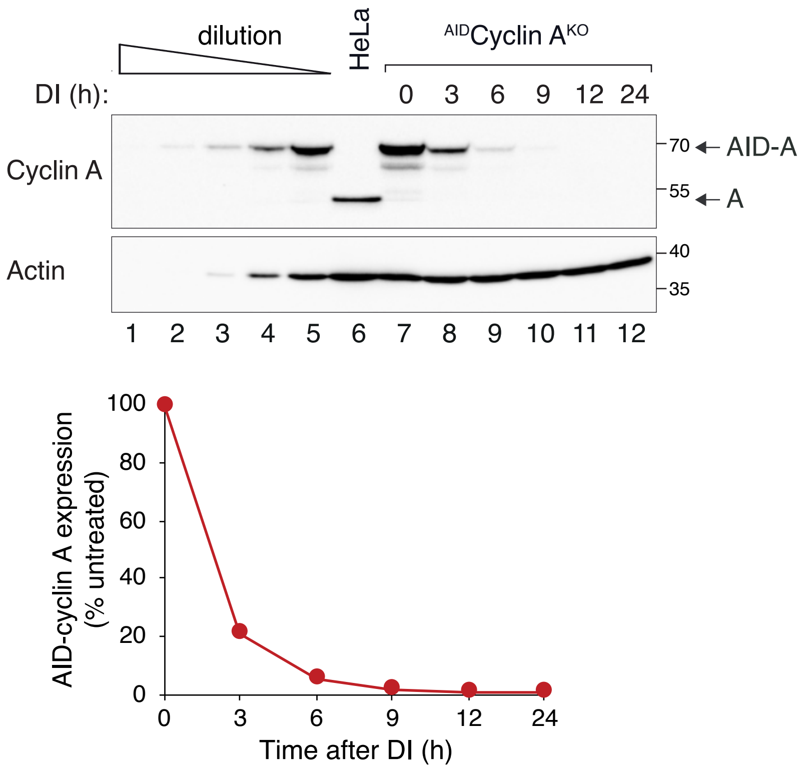

Supplemental Figure S1

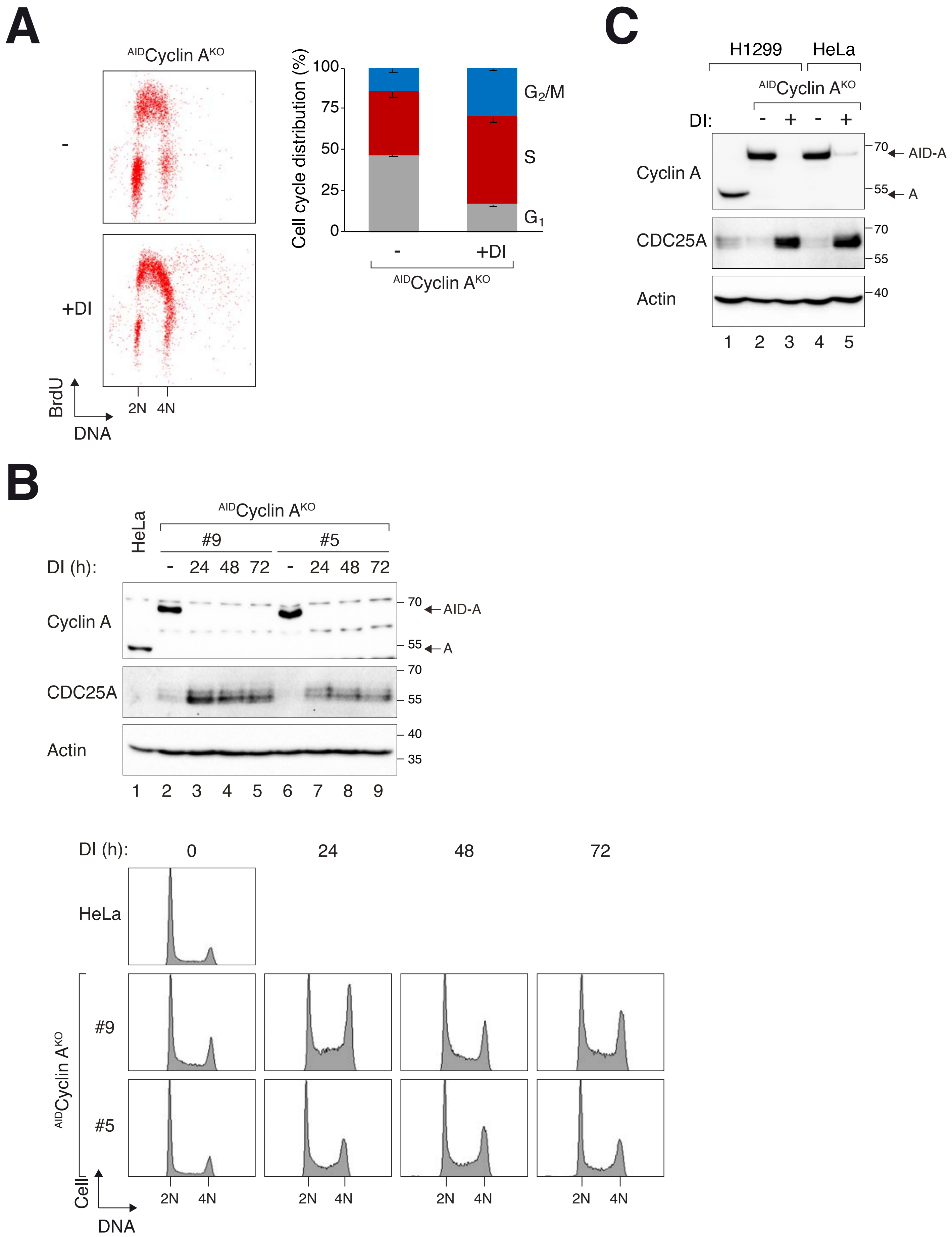

Supplemental Figure S2

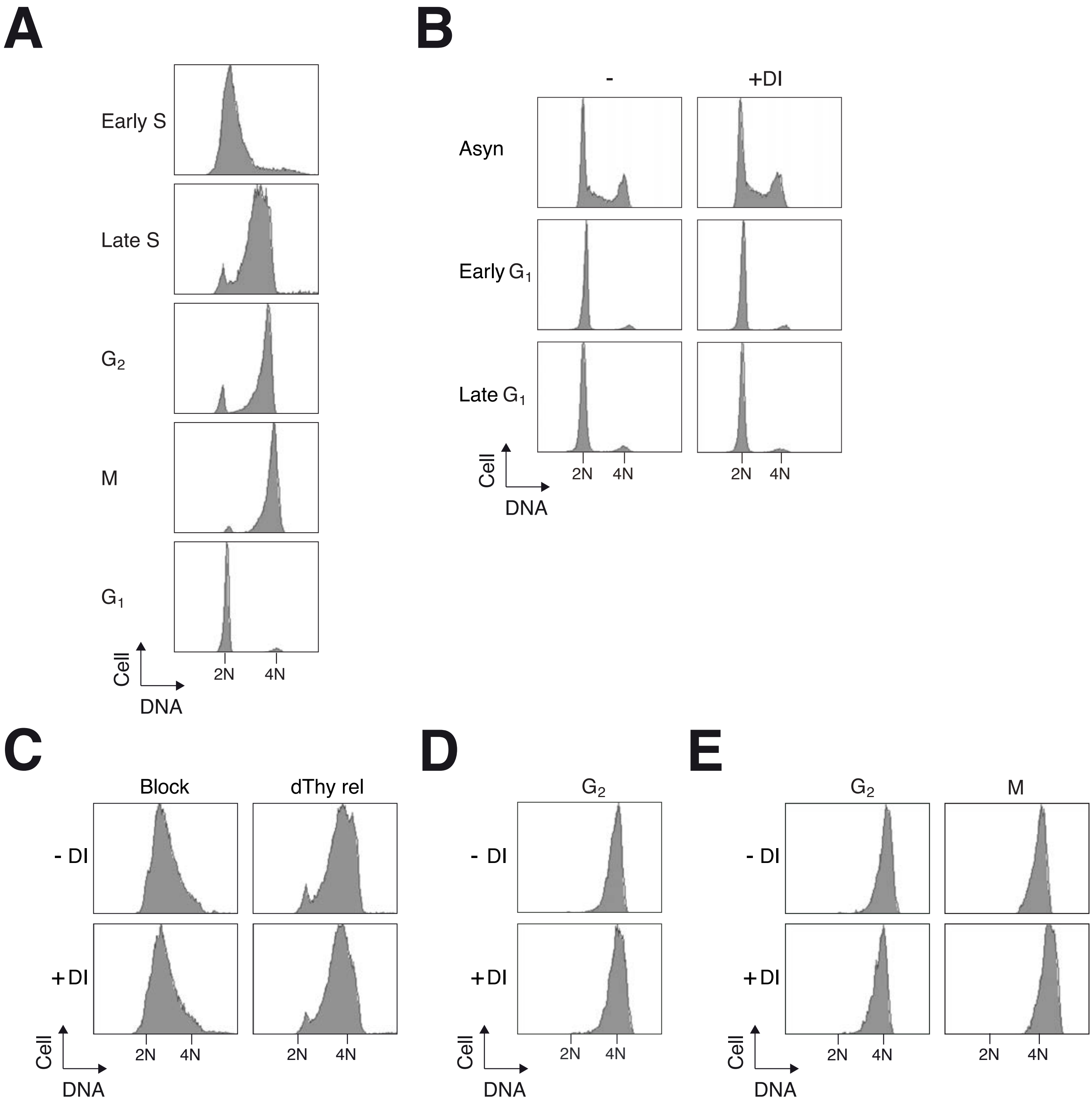

Supplemental Figure S3

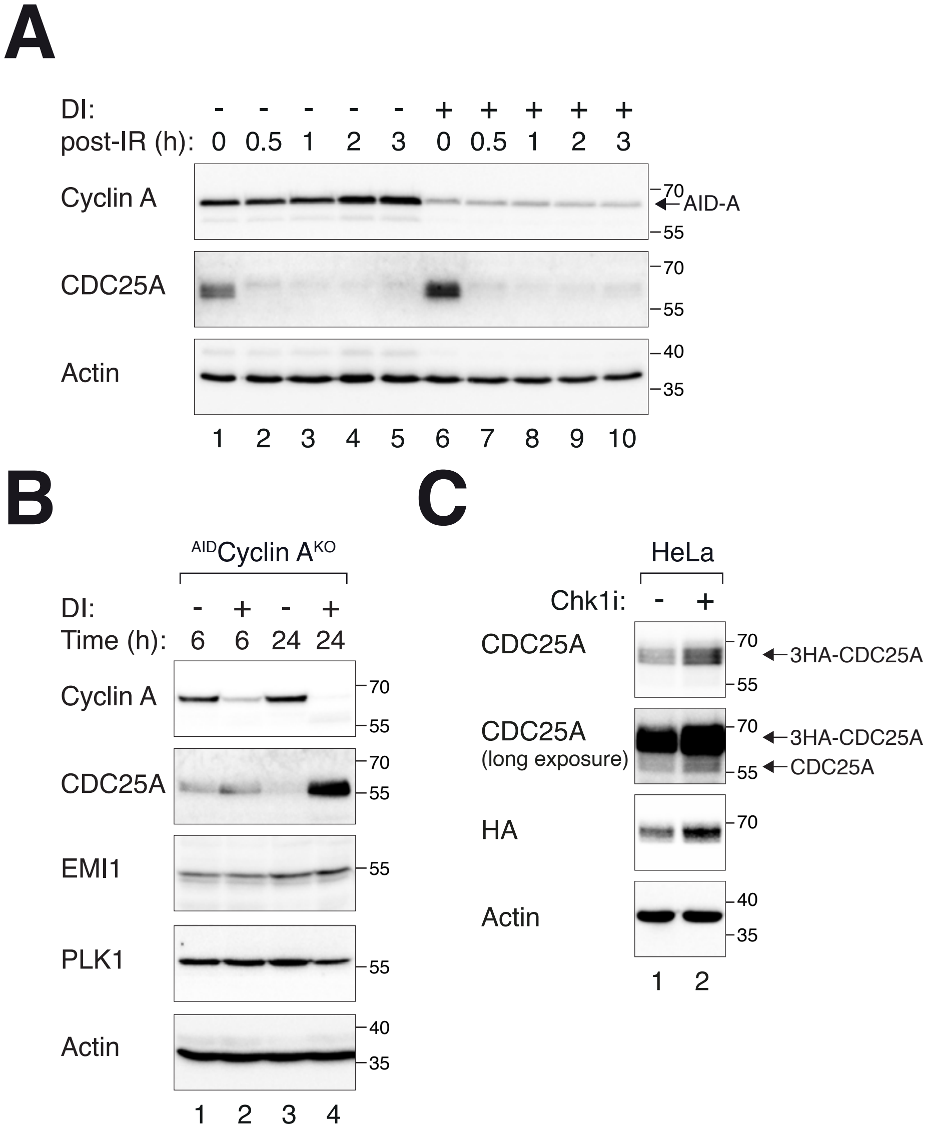

Supplemental Figure S4

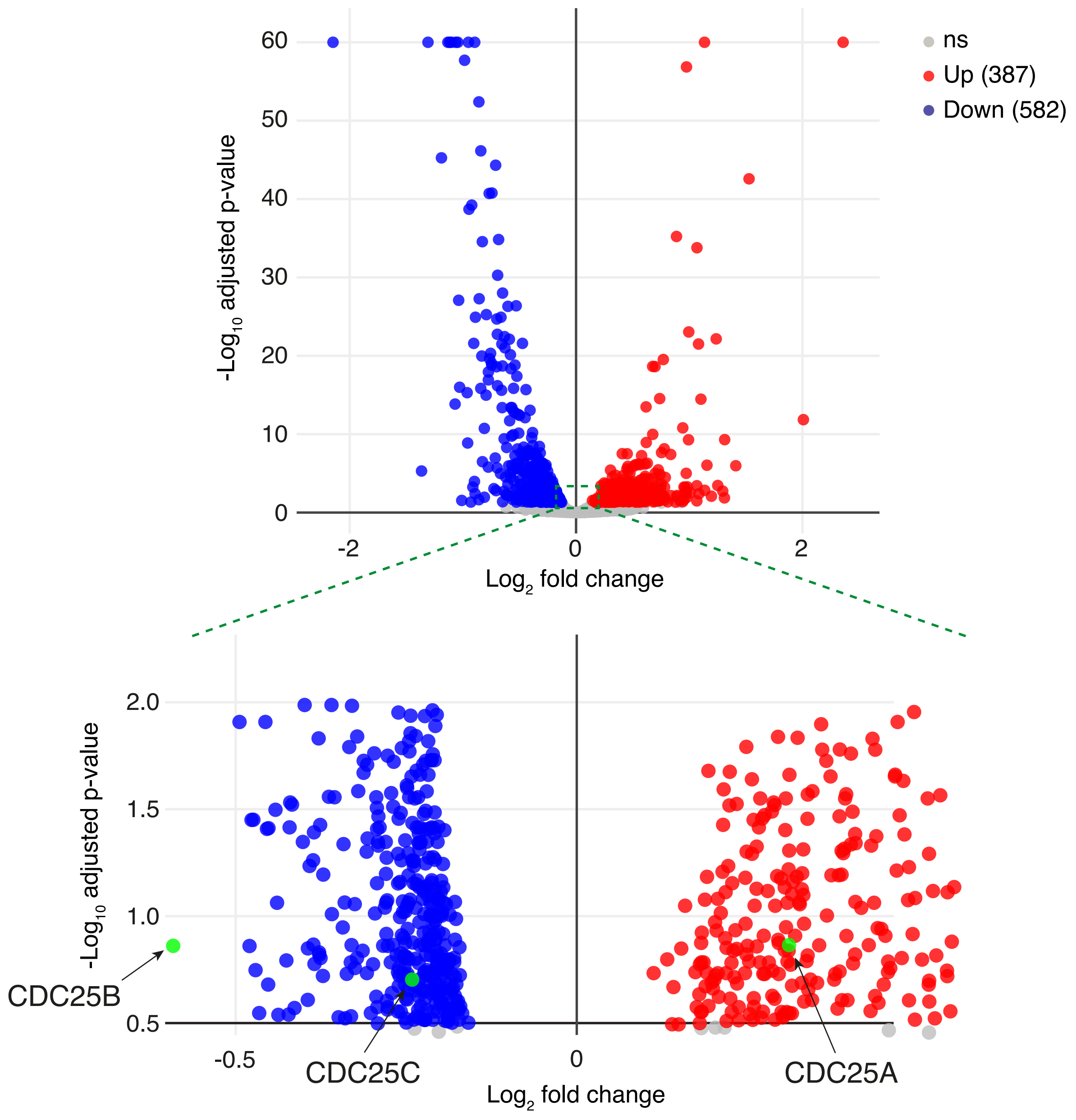

Supplemental Figure S5

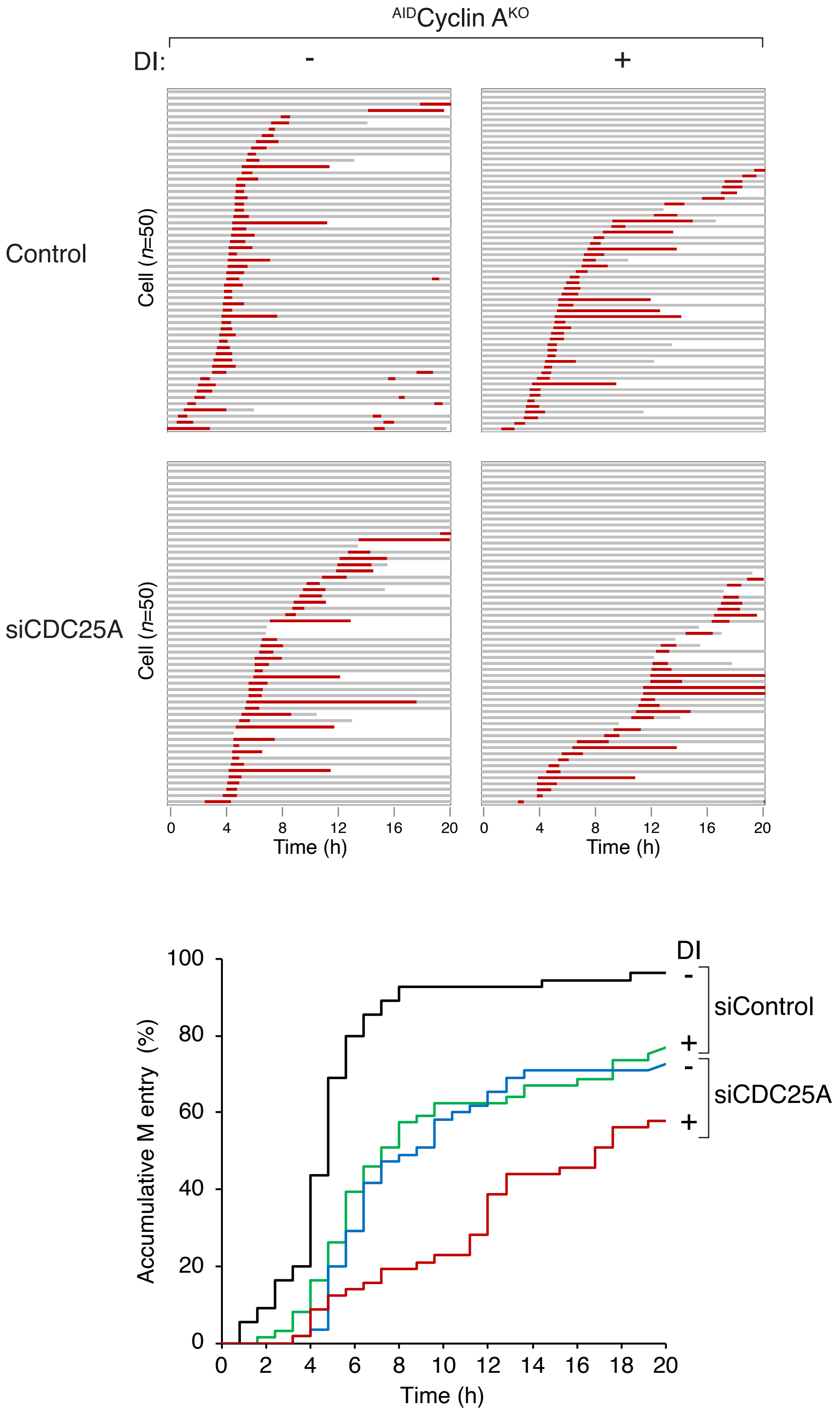

Supplemental Figure S6

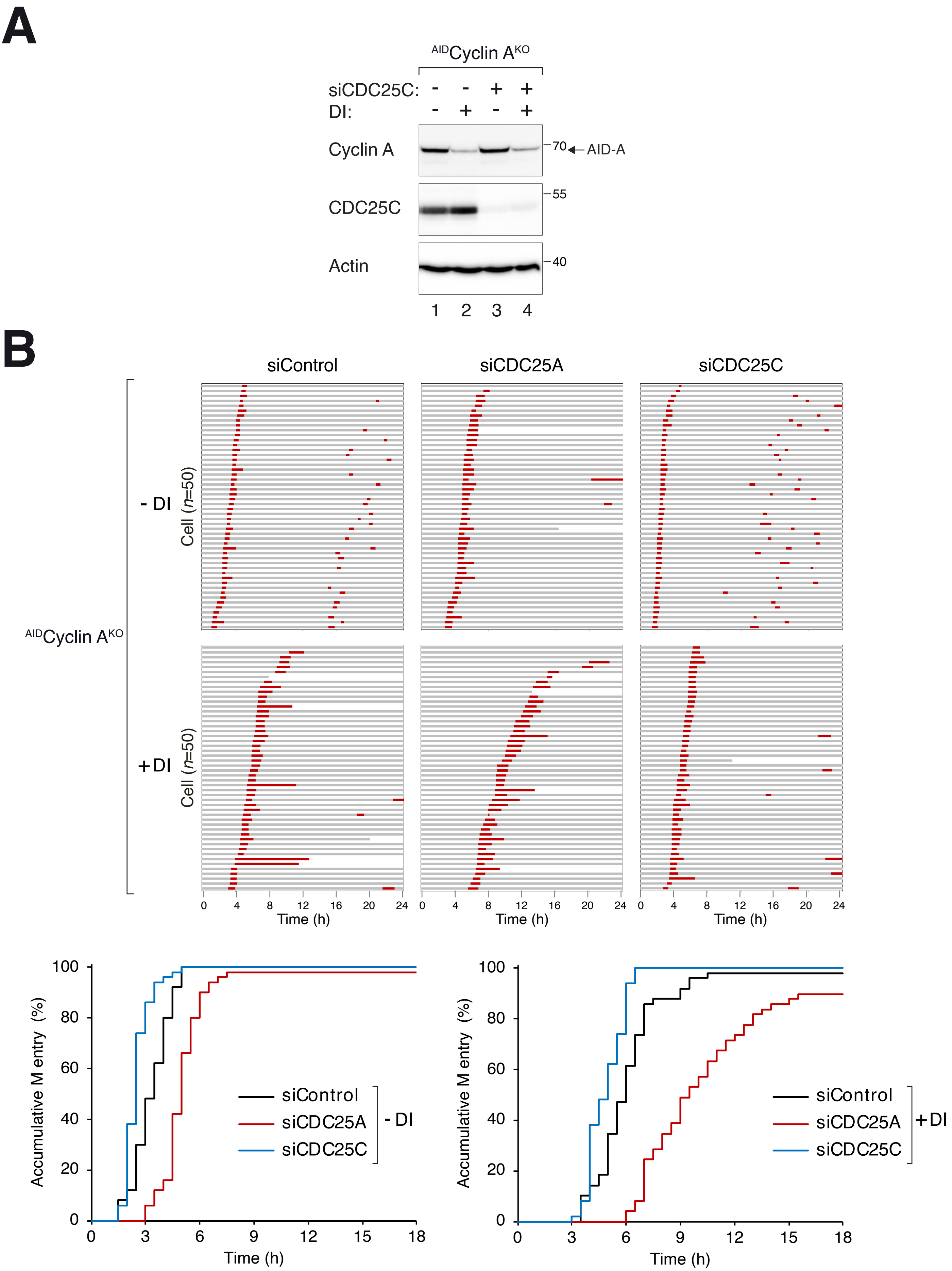

Supplemental Figure S7
